# 

**Published:** 1919-12-06

**Authors:** 


SPECIAL NUMBER.

### THE HOSPITAL

**The Modern Newspaper of Administrative Medicine and Institutional Life.**

Telegraphic Address: OSPEDALE, RAND, LONDON. Telephone Number: 2734 GERRARD.

No. 1748, Vol. LXVII. | Saturday, December 6th, 1919. | PRICE TWOPENCE

